# Application of a Genus-Specific LAMP Assay for Schistosome Species to Detect *Schistosoma haematobium* x *Schistosoma bovis* Hybrids

**DOI:** 10.3390/jcm10061308

**Published:** 2021-03-22

**Authors:** Beatriz Crego-Vicente, Pedro Fernández-Soto, Begoña Febrer-Sendra, Juan García-Bernalt Diego, Jérôme Boissier, Etienne K. Angora, Ana Oleaga, Antonio Muro

**Affiliations:** 1 Infectious and Tropical Diseases Research Group (e-INTRO), Research Centre for Tropical Diseases at the University of Salamanca (IBSAL-CIETUS), Biomedical Research Institute of Salamanca, Faculty of Pharmacy, University of Salamanca, 37007 Salamanca, Spain; beatrizcregovic@usal.es (B.C.-V.); begofebrer@usal.es (B.F.-S.); juanbernalt95@usal.es (J.G.-B.D.); 2 IHPE, Université Montpellier, CNRS, Ifremer, Université Perpignan Via Domitia, 66100 Perpignan, France; boissier@univ-perp.fr; 3 Swiss Tropical and Public Health Institute, P.O. Box CH-4002 Basel, Switzerland; kpongboetienne.angora@swisstph.ch; 4 Department of Public Health, University of Basel, P.O. Box CH-4003 Basel, Switzerland; 5 Unité de Formation et de Recherche Sciences Pharmaceutiques et Biologiques, Université Félix Houphouët-Boigny, Abidjan BPV 34, Côte d’Ivoire; 6 Parasitology Laboratory, Institute of Natural Resources and Agrobiology (IRNASA, CSIC), Cordel de Merinas 40-52, 37008 Salamanca, Spain; ana.oleaga@irnasa.csic.es

**Keywords:** LAMP, schistosomiasis, schistosome hybrids, *Schistosoma haematobium*, *Schistosoma bovis*, molecular diagnosis, species-specific LAMP, genus-specific LAMP

## Abstract

Schistosomiasis is a disease of great medical and veterinary importance in tropical and subtropical regions caused by different species of parasitic flatworms of the genus *Schistosoma*. The emergence of natural hybrids of schistosomes indicate the risk of possible infection to humans and their zoonotic potential, specifically for *Schistosoma haematobium* and *S. bovis*. Hybrid schistosomes have the potential to replace existing species, generate new resistances, pathologies and extending host ranges. Hybrids may also confuse the serological, molecular and parasitological diagnosis. Currently, LAMP technology based on detection of nucleic acids is used for detection of many agents, including schistosomes. Here, we evaluate our previously developed species-specific LAMP assays for *S. haematobium*, *S. mansoni*, *S. bovis* and also the genus-specific LAMP for the simultaneous detection of several *Schistosoma* species against both DNA from pure and, for the first time, *S. haematobium* x *S. bovis* hybrids. Proper operation was evaluated with DNA from hybrid schistosomes and with human urine samples artificially contaminated with parasites’ DNA. LAMP was performed with and without prior DNA extraction. The genus-specific LAMP properly amplified pure *Schistosoma* species and different *S. haematobium-S. bovis* hybrids with different sensitivity. The *Schistosoma* spp.-LAMP method is potentially adaptable for field diagnosis and disease surveillance in schistosomiasis endemic areas where human infections by schistosome hybrids are increasingly common.

## 1. Introduction

Environmental changes due to ecosystem decline, biodiversity loss and climate change are some issues with potential ecological risk that we are facing as human beings. These changes driven by increasing economic development, migration, agricultural and livestock practices and deforestation have consequences in emerging infectious diseases (EIDs) [1,2,3,4,5,6]. Changes in biodiversity have the potential to either increase or reduce the incidence of infectious disease in humans because they involve interactions among species. The appearance of diseases in non-endemic areas and the increase in encounters between different species, when ecological and geographic barriers are lost, lead to the emergence of new hybrid forms [4]. Hybridization of parasites is an emerging public health issue [6], mainly because hybrid forms have the potential to generate new resistances, pathologies, be more virulent as well as affect new hosts [4,6].

Schistosomiasis is one of the most important parasitic diseases of humans in terms of morbidity and mortality, ranking secondary to malaria. The World Health Organization (WHO) estimates that almost 240 million people are affected worldwide (up to 90% in Africa) with 700 million people living in tropical and subtropical endemic areas in over 78 countries [7,8,9]. The parasitic flatworms responsible of schistosomiasis are digenetic trematodes worms of the genus *Schistosoma* that infect both humans and animals. The three main species infecting humans are *S. haematobium*, *S. mansoni* (both in Africa and the Middle East; *S. mansoni* is also present in the Americas) and *S. japonicum* (Asia). Other schistosome species have been linked to human infections, including *S. intercalatum*, *S. guineensis* (both in West and Central Africa), *S. mekongi* (in Kong Island) [10,11] and *S. malayensis* (Malaysia) [12], or with potential to infect humans, such us *S. mattheei* (in Africa) [13]. Livestock schistosomiasis due to *S. bovis*, *S. curassoni* and *S. mattheei* in cattle, sheep and goats is a common parasitic infection in sub-Saharan Africa, and it is an important cause of animal mortality and morbidity [5,14]. The emergence of natural hybrids of *S. haematobium-S. guineensis* [15], *S. haematobium-S. intercalatum* [16,17], *S. haematobium-S. mattheei* [18] and mainly *S. haematobium* and the cattle schistosome *S. bovis* [2,19,20,21,22] clearly indicate the risk of hybrids that can potentially infect humans and their zoonotic potential [23]. *Schistosoma bovis* is one of the most significant veterinary problems in Africa [24,25] and to date is considered as a possible emerging health threat after the molecular characterization of *S. haematobium-S. bovis* hybrids from children in Senegal [2], in Côte d’Ivoire [26], in Benin [23], in Niger [27], in Mali [28] and in a schistosomiasis outbreak in Corsica, France [19]. *S. bovis* is phylogenetically a close relative of *S. haematobium*, and their close relationship and overlapping geographical distribution allows these to hybridize in the wild, increasing their genetic diversity and the risk of zoonotic transmission from animal reservoirs to humans [22]. Moreover, zoonotic hybrids could replace existing species and parasite strains extending intermediate and definitive host ranges, complicating transmission or presenting and increasing infectivity and virulence [6]. Furthermore, hybrid schistosomes forms may confuse the serological, molecular and, especially, parasitological diagnostic because the presence of excreted ova with atypical morphology [29,30].

However, in endemic countries schistosomiasis is definitively diagnosed by microscopic examination of excreted eggs in stool (*S. mansoni*, *S. japonicum*, *S. intercalum*, *S. guineensis* and *S. mekongi*) by the Kato-Katz method (KK) or in urine (*S. haematobium*) by filtration or sedimentation techniques. Typically, microscopy is relatively time-consuming and lacks in sensitivity, mainly in areas with low-intensity infections [7,31,32]. Numerous serological diagnostic approaches, including enzyme-linked immunosorbent assay (ELISA) and indirect hemagglutination (IHA) tests, in addition to other assays based on the antibody and antigen detection have been widely evaluated, but cross-reactivity, differences in sensitivity and a lack of standardization have been reported [33,34,35]. To try to solve these disadvantages, a large number of more sensitive and accurate PCR-based molecular methods have also been developed both for the diagnosis of human and animal schistosomiasis [36], being especially valuable in simultaneous detection and identification of *Schistosoma* species [37]. However, the complex PCR-based techniques are expensive and difficult to apply routinely in field conditions in endemic areas of schistosomiasis. In this sense, loop-mediated isothermal amplification (LAMP) technology [38] has been recently revealed as a versatile alternative, having great potential for molecular diagnosis in limited-resource settings in endemic areas [39,40]. To date, a number of LAMP approaches have been developed to detect specifically *S. haematobium*, *S. mansoni* and *S. japonicum* in urine, stool, and snails specimens, as recently summarized by Avendaño and Patarroyo [41]. In addition, a novel species-specific LAMP to detect *S. bovis* and a genus-specific LAMP to detect different *Schistosoma* species (including *S. haematobium*, *S. intercalatum*, *S. mansoni* and *S. bovis*) have been recently reported by our group [42]. Due to the schistosome hybridization rapid emergence and spread, and the consequences for disease prevalence, pathological characteristics and treatment, a LAMP test to detect several *Schistosoma* species (including hybrids forms) would be very useful for the diagnosis and management of schistosomiasis.

Thus, in this study we examined the utility of our recently developed genus-specific LAMP assay to detect *Schistosoma* species in the detection of different hybrid schistosome molecular profiles. Moreover, we evaluated the *Schistosoma* spp.-LAMP in simulated human urine samples spiked with serially diluted DNA from hybrid specimens using both urine with and without prior DNA extraction.

## 2. Materials and Methods

### 2.1. Schistosoma Species DNA Samples

Genomic DNA (gDNA) samples from several hybrid schistosomes (miracidia) and pure adults *S. haematobium*, *S. mansoni*, and *S. bovis* species were used in our study. The gDNA of schistosome hybrids was obtained from parasites collected in previous studies carried out in Agboville (Côte d’Ivoire) by Angora et al. [26] and in Corsica, France, by Boissier et al. [19]. The schistosome hybrids profiles, according to the rapid diagnostic mitochondrial cox1 analysis and by sequencing of the cox1 and ITS regions, respectively, as described elsewhere [26], are specified in Table 1. Pure *S. haematobium* gDNA (Egyptian strain) was kindly provided by the Laboratoire Interactions Hôtes-Pathogènes-Environnements (IHPE), University Perpignan Via Domitia, Perpignan, France. The laboratory strain from Egypt is experimentally maintained in the culturing facilities at the University of Perpignan and was originally provided by the Biomedical Research Institute, Rockville, Maryland [43]. *S. bovis* was provided by the laboratory of Animal Parasitology of the Institute of Natural Resources and Agrobiology of Salamanca where it has been maintained in hamsters and sheep experimentally infected. *S. mansoni* is maintained by serial passages in mice routinely infected in the Laboratory of Parasitic and Molecular Immunology, CIETUS, University of Salamanca, Salamanca, Spain. *S. mansoni* gDNA (Brazilian strain) and *S. bovis* gDNA (Spanish strain) were obtained from frozen adult male and female worms using the NucleoSpin Tissue Kit (Macherey-Nagel, GmbH & Co., Dueren, Germany) following the manufacturers’ instructions. All DNA samples were measured using a Nanodrop ND-100 spectrophotometer (Nanodrop Technologies) and then diluted to final 20 ng/µL and 10 ng/µL concentrations. Subsequently, from 10 ng/µL concentration serial 10-fold dilutions were prepared with ultrapure water ranging from 10^−1^ to 10^−6^ and stored at −20 °C until use.

### 2.2. Urine Samples Spiked with gDNA from Schistosoma Species

Fresh urine was collected from healthy staff donors with no history of travel to endemic areas of schistosomiasis to assess both specificity and sensitivity of the *Schisto*-LAMP assays. Urine was divided into aliquots of 100 μL each and then artificially spiked with 2 μL of 10-fold serially diluted gDNA from *Schistosoma* species ranging from 20 ng/μL to 100 fg/μL, thus resulting in a set of artificial urine samples with a final hybrid schistosomes gDNA concentration ranging from 0.8 ng/μL to 4 fg/μL. These fresh simulated urine samples were prepared when required and analyzed in *Schisto*-LAMP assays following two procedures. In the first procedure, we used the “Rapid-Heat LAMP method” as described elsewhere by Gandasegui et al. [44]. In brief, each aliquot of urine was heated at 95 °C for 15 min and shortly spun to pellet the debris. Subsequently, 2 μL of the supernatant was used directly as template for LAMP reactions. After analysis, the remaining volume of each aliquot was stored at −20 °C. In the second procedure, the frozen simulated urine samples were thawed and DNA was extracted using the i-genomic Urine DNA Extraction Mini Kit (Intron Biotechnology, UK) following the manufacturers’ instructions. DNA obtained from aliquots was stored at −20 °C until use in a second LAMP screening.

### 2.3. Schisto-LAMP Assays

LAMP assays were accomplished using the reaction mixtures and specific primer sets previously described elsewhere by our group for detection of species-specific *S. mansoni* based on a mitochondrial minisatellite DNA region [45], *S. haematobium*, based on the ribosomal intergenic spacer (IGS) [44], and *S. bovis*, based on the mitochondrial NADH subunit 1 [42]. A genus-specific LAMP assay designed on the internal transcribed spacer 1 (ITS-1) for the simultaneous detection of different species, including *S. mansoni*, *S. haematobium*, *S. intercalatum* and *S. bovis*, was also applied [42]. The reactions were carried out using previously described conditions, with the exception of the final reaction volume, which was reduced from 25 μL to 15 μL. Briefly, LAMP reaction mixtures (15 μL) contained 40 pmol each of FIP and BIP primers, 5 pmol each of F3 and B3 primers, 0.4 μM of each LB and LF primers (if applicable) (Table 2), 1.4 mM of each dNTP (Bioron), 1x Isothermal Amplification Buffer—20 mM Tris-HCl (pH 8.8), 50 mM KCl, 10 mM (NH_4_)_2_SO_4_, 2 mM MgSO_4_, 0.1% Tween20 (New England Biolabs Ltd., Ipswich, MA, USA)—supplementary with 6 mM MgSO_4_ and 8 U of *Bst* 2.0 WarmStart DNA polymerase (New England Biolabs Ltd., Ipswich, MA, USA) with 2 μL of template. *Schisto*-LAMP reactions were performed in 0.5 mL tubes that were incubated in a heat block at 65 °C for 60 min and then heated at 80 °C for 5–10 min to stop the reaction.

### 2.4. Specificity and Sensibility of Schisto-LAMP Assays in Detecting Schistosome Hybrids

The specificity of *Schisto*-LAMP assays to amplify both pure *Schistosoma* species (*S. haematobium, S. mansoni* and *S. bovis*) and hybrids was tested against parasite DNA samples used as controls, as mentioned above. To determine the lower detection limit of the *Schisto*-LAMP assays, gDNA from hybrid schistosomes 10-fold serially diluted was used as template for amplification. Moreover, the sensitivity was also assayed with the simulated urine samples artificially spiked with hybrid schistosomes gDNA both without prior DNA extraction and after DNA extraction by using the commercial kit.

### 2.5. Detection of LAMP Products

LAMP results were visually detected by the naked eye by adding 2 μL (1:10, 10,000x) SYBR Green I fluorescent dye (Invitrogen, Carlsbad, California, USA) to each reaction tube post-amplification. Green fluorescence was observed in LAMP-positive reactions and original orange in LAMP-negative reactions. In addition, the LAMP products (3–5 μL) were visualized by Midori Green Advance DNA (Nippon Genetics Europe GmbH, Dueren, Germany) staining in 1.5% agarose gels to corroborate the colorimetric results. The LAMP amplifications showed a characteristic ladder-like band pattern.

## 3. Results

### 3.1. Schisto-LAMP Assays Performance

**Figure 1:**
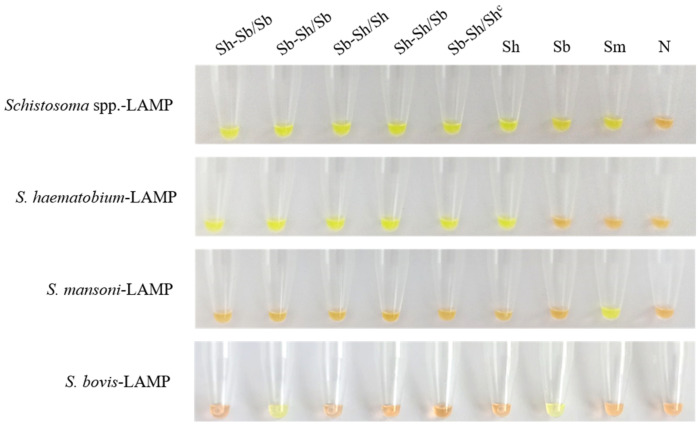
*Schisto*-LAMP assays performance in testing DNA samples from both pure *Schistosoma* species and hybrids. *Schistosoma* spp.-LAMP, the genus-specific LAMP for detecting several schistosome species; *S. haematobium*-LAMP, *S. mansoni*-LAMP and *S. bovis*-LAMP, the species-specific LAMP assays for detecting *S. haematobium*, *S. mansoni* and *S. bovis*, respectively. Sh-Sb/Sb, Sb-Sh/Sb, Sb-Sh/Sh, Sh-Sh/Sb, DNA from schistosomes hybrids from Agboville. Sb-Sh/Shc, corsican hybrid schistosome. Sh, Sb, Sm, DNA from pure *S. haematobium*, *S. bovis* and *S. mansoni*, respectively. N, negative control (no DNA template).

The results obtained in testing the different *Schisto*-LAMP assays against both the hybrid schistosomes gDNA and pure *Schistosoma* species used as controls are shown in Figure 1. All DNA samples tested positive by the genus-specific LAMP. The species-specific LAMP for detecting *S. haematobium* amplified all DNA samples with the exception of *S. bovis* and *S. mansoni* DNA. As expected, the species-specific LAMP for *S. mansoni* only amplified DNA from this parasite. Finally, the species-specific LAMP for *S. bovis* amplified both pure *S. bovis* DNA and *S. bovis-S. haematobium* x *S. bovis* hybrid parasite DNA.

### 3.2. Sensitivity of Genus-Specific-LAMP Assay in Detection of Hybrid Schistosomes

**Figure 2:**
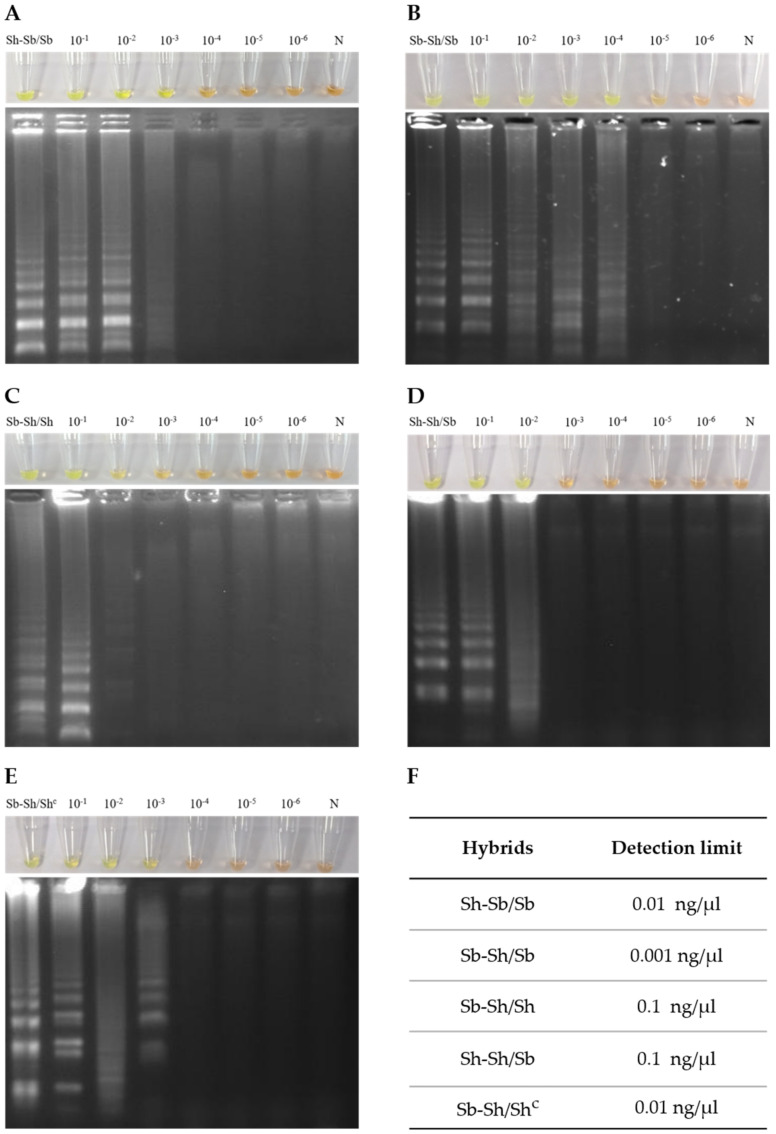
Assessment of genus-specific LAMP analytical sensitivity for hybrid schistosomes using gDNA serial dilutions. The figure shows the genus-specific LAMP results by color change (top) and in agarose electrophoresis (bottom) for each hybrid schistosome tested: (**A**) Sh-Sb/Sb; (**B**) Sb-Sh/Sb; (**C**) Sb-Sh/Sh; (**D**) Sh-Sh/Sb and (**E**) Sb-Sh/Sh^c^). (**F**) Summary table indicating the genus-specific LAMP detection limit for detecting each hybrid. Lanes 10^−1^–10^−6^: 10-fold serial dilutions. N, negative control (no DNA template).

The genus-specific LAMP assay detection limit was different for the different hybrid schistosomes tested (Figure 2). The hybrids Sb-Sh/Sh and Sh-Sh/Sb DNA amplification detection limit was 0.1 ng/µL, whereas the hybrids Sh-Sb/Sb and Sb-Sh/Sh (Corsican hybrid) detection limit was 0.01 ng/µL. The lowest limit of detection was 0.001 ng/µL for hybrid Sb-Sh/Sb.

### 3.3. Detection Limit of Genus-Specific LAMP Assay in Simulated Human Urine Samples

**Figure 3:**
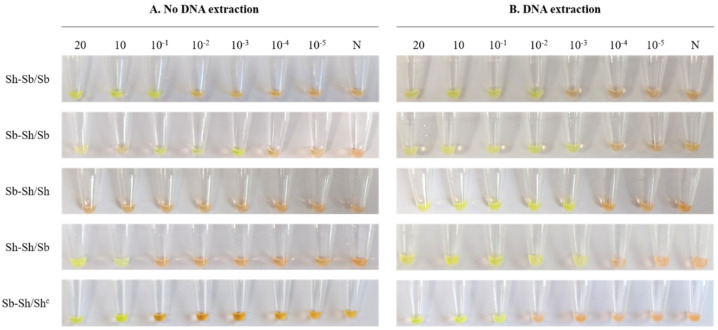
Sensitivity of the genus-specific LAMP assay in simulated human urine samples artificially spiked with gDNA from different hybrid schistosomes. (**A**) Sensitivity assessment of LAMP when performing a simple heating method from serial dilutions of hybrid schistosomes gDNA. (**B**) Sensitivity assessment of LAMP when performing the DNA extraction with the i-genomic Urine DNA Extraction Mini Kit (Intron Biotechnology, UK) from serial dilutions of hybrid schistosomes gDNA. Lanes 20, 10 and 10^−1^–10^−5^: 20 ng, 10 ng and 10-fold serial dilutions, respectively; Sh-Sb/Sb, Sb-Sh/Sb, Sb-Sh/Sh, Sh-Sh/Sb, and Sb-Sh/Sh^c^: gDNA from hybrid schistosomes; N: negative controls (no DNA template).

The detection limit of genus-specific LAMP for hybrid schistosomes in simulated urine samples spiked with serial dilutions of gDNA is shown in Figure 3. As observed by colorimetric change, the sensitivity was generally lower when using the urine samples without prior extraction of DNA (for Sh-Sb/Sb: 0.1 ng/µL; Sh-Sh/Sb and Sb-Sh/Shc: 1 ng/µL) than when using a commercial kit for extraction (for Sh-Sb/Sb: 0.01 ng/µL; Sh-Sh/Sb: 0.001 ng/µL and Sb-Sh/Sh^c^: 0.1 ng/µL). It should be noted that we obtained the same detection limit of genus-specific LAMP assay for hybrid Sb-Sh/Sb using both procedures for analysis (0.001 ng/µL). Unexpectedly, hybrid Sb-Sh/Sh did not amplify using the simple heat method but did when a prior DNA extraction was carried out, reaching a limit of detection of 0.001 ng/µL.

## 4. Discussion

*Schistosoma* species hybridization in nature is an emergent issue for public health [4,6]. Molecular data from hybridizations between schistosome collections have identified new species distributions [46], interspecies both human-specific and animal-specific hybridization [22,47,48,49], and surprising host associations and multi-host transmission [23,50,51]. Molecular detection of the hybrid schistosomes adds a new perspective to the diagnosis, epidemiology and control of schistosomiasis.

In this work, we tested our previously developed species-specific LAMP assays for *S. haematobium* [44], *S. mansoni* [45], *S. bovis* and also the genus-specific LAMP for the simultaneous detection of several *Schistosoma* species [42] against both gDNA from pure and, for the first time, hybrid schistosomes. These hybrids were obtained in studies conducted in Côte d’Ivoire [26] and Corsica, France [19], and subsequently well characterized by amplification and sequencing of a partial fragment of the mitochondrial cytochrome c oxidase subunit 1 (cox1) and the complete nuclear ribosomal DNA internal transcribed spacer (ITS).

As expected, in our trials, species-specific LAMP for *S. mansoni* only amplified DNA from pure parasite but not from other schistosomes nor hybrids *S. bovis-S. haematobium* or *S. haematobium-S. bovis*, thus corroborating again its high specificity in detection of *S. mansoni*. The *S. mansoni*-LAMP was originally designing on a 620 bp sequence corresponding to a specific mitochondrial *S. mansoni* minisatellite DNA region [52] and has been already specifically tested by our group in stool samples from experimentally infected mice [45], in both human stool and snail samples in field conditions [53], and also in human urine samples [54]. Notwithstanding, it should be very interesting to assess this *S. mansoni*-LAMP in *S. mansoni-S. haematobium* hybrid parasites detection since these hybrid forms have been already described in a study carried out in schoolchildren from northern Senegal [49] and, more recently, in a migrant boy from Côte d’Ivoire entering France [55].

The species-specific LAMP for *S. bovis* amplified gDNA from pure *S. bovis* but not from *S. haematobium* nor *S. mansoni*, showing its high specificity in detecting only that species. The two hybrids with a *S. haematobium* mitochondrial (cox1) profile (Sh-Sb/Sb and Sh-Sh/Sb) did not amplify. Unexpectedly, among those hybrids with a *S. bovis* mitochondrial (cox1) profile (Sb-Sh/Sb, Sb-Sh/Sh, and Sb-Sh/Sh^c^), the only one amplified was *S. bovis-S. haematobium/S. bovis* but not those with a *S. haematobium* double-banded ITS rDNA profile. Our *S. bovis*-LAMP is based on a 678 bp sequence derived from mitochondrial NADH subunit 1 (NADH-1) first reported by Xiao et al. (2010). Sequences generated from the mitochondrial (mt) DNA, including cox1 and NADH 1, are the most commonly used mitochondrial markers for studies on flatworms helping to establish the population and genetic relationship among *Schistosoma* species [56]. mtDNA is usually maternally inherited in almost all metazoans and is considered to be clonal and rarely or never undergoes recombination. Nevertheless, mtDNA rapidly accumulate mutations over time and shows a higher level of divergence among species relative to intra-specific variation [47,57]. This could be a possible explanation for lack of amplification in both hybrid Sb-Sh/Sh (from Côte d’Ivoire) and Sb-Sh/Sh^c^ (from Corsica) profiles. The fact that only the hybrid Sb-Sh/Sb was amplified by *S. bovis*-specific LAMP may be interpreted because the hybrid line is the result of an initial cross between a male *S. haematobium* and a female *S. bovis*, leading to introgression of *S. bovis* mtDNA into *S. haematobium* [2], most likely favoring the amplification of the *S. bovis* profile.

As expected, the genus-specific LAMP achieved DNA amplification of pure *Schistosoma* species (*S. mansoni, S. haematobium* and *S. bovis*) as verified previously by our group [42], but also, and very interestingly, all hybrid schistosomes tested. The *Schistosoma* spp.-LAMP design is based on a 457 bp ITS-1 sequence type from *S. haematobium* [58]. ITS-1 and ITS-2 are sequences of non-functional RNA situated between structural ribosomal RNAs on a common precursor transcript. ITS-2 has been widely used in trematode identification because it is usually conserved within species but more variable among species [59,60]. The schistosome ITS-2 is particularly powerful marker to detect introgression. This region can retain both parental copies for several generations before they are homogenized by concerted evolution, the nuclear DNA profiles resulting in double chromatogram peaks at the species-specific mutation sites [6]. On the other hand, the schistosome ITS-1 contains an original main repeating sequence unit from the 3’ end of the 18S rRNA gene which, in turn, contains a sub-repeat that varies slightly in size and composition [61]. There is a high degree of sequence conservation between the repeats, but variation in sequence patterns and their number occur both within and between species. For example, the ITS-1 region of *S. haematobium* contains two tandemly repeated elements, whereas *S. japonicum* group of species contains as many as seven repeats [62]. In general, multiple repeats and intra-individual variation in numbers and abundance of these is a feature of the Asian schistosomes (*S. japonicum* and *S. indicum* groups), but not generally of African schistosomes (*S. mansoni* and *S. haematobium* groups), in which an absence of intra-individual variation in the ITS-1 was reported [63]. In this regard, since the hybrid schistosomes tested has been molecularly characterized by analysis of mitochondrial cox1 as *S. haematobium/S. bovis* species designation (hence, African schistosomes), our genus-specific LAMP could detect the hybrids probably because of that lack of intra-individual variation in the ITS-1 type targeted sequence. Additionally, processes such as hybridization could cause the sharing of different ITS types among *Schistosoma* species [57], which would likely affect the sensitivity in detecting gDNA from the different hybrid specimens. In this sense, using *Schistosoma* spp-LAMP, we previously reported a limit of detection for pure *S. haematobium* and *S. bovis* species of 0.1 pg and 10 pg, respectively [42]; however, a lower sensitivity ranging from 100 pg to 1 pg was now obtained when testing the different crossing of *S. haematobium* and *S. bovis* species. Interestingly, for hybrid Sb-Sh/Sb a limit of detection 10 times higher than for pure *S. bovis* was obtained (1 pg vs. 10 pg). Despite the variation in sensitivity of the *Schistosoma* spp.-LAMP between the detection of pure *Schistosoma* species and hybrid forms, it is very important to highlight the possibility of amplifying both pure and hybrid schistosomes in order to use LAMP as a single molecular tool for the diagnosis and surveillance of schistosomiasis, mainly in endemic areas of the disease. As mentioned above for *S. mansoni*-LAMP, it would also be very interesting to test if the *Schistosoma* spp.-LAMP assay (that detects pure *S. mansoni*) could amplify the more surprising hybridization between *S. mansoni* and *S. haematobium* that infect humans [6,55].

Similarly remarkably, the species-specific LAMP for *S. haematobium* amplified all gDNA from hybrid forms in addition to the pure *S. haematobium* gDNA. Our *S. haematobium*-LAMP is based on a 2522 bp sequence of *S. haematobium* ribosomal intergenic spacer (IGS) DNA (GenBank: AJ223838) [64] that amplifies a highly specific sub-sequence target of 199 bp of that species [44]. The IGS of *Schistosoma* species contains many repeats, and recombination is a relatively frequent event, although sequences of *S. haematobium* are well conserved within the IGS [64]. Since the molecular characterization of the hybrids showed a *S. haematobium* signature (either by cox1 or ITS-2 genetic profiles), amplification of the *S. haematobium* IGS sequence could be possible in all hybrid schistosomes tested.

Regarding urine samples analysis, we are aware that our *Schistosoma* spp.-LAMP has not been tested with clinical specimens, but results obtained in simulated human urine samples indicate that, although with some differences, the LAMP test is sensitive enough to detect hybrid schistosomes at a low level in urine. Better results were obtained when applying a commercial kit for DNA extraction than heated urine, because of the well-known effectiveness of this procedure to isolate genomic DNA from urine samples suitable for further molecular analyses [65]. However, amplification (with the unexplainable exception in hybrid Sb-Sh/Sh) was also obtained just with heated whole urine without prior DNA extraction at an acceptable level. This inexpensive and simple rapid-heating procedure could be potentially very useful under certain circumstances when a large number of samples must be tested, mainly in low-resource settings in endemic areas.

## 5. Conclusions

In conclusion, the results of this preliminary study demonstrated that the genus-specific LAMP assay could be a potential molecular tool to be use for detection, not only for different pure schistosome species, but also for hybrids *S. haematobium-S. bovis* in urine samples. Although further research for evaluation of the assay for the application in clinical samples is required, the method is potentially adaptable for field diagnosis and disease surveillance in schistosomiasis endemic areas where human infections by schistosome hybrids are increasingly common.

## Figures and Tables

**Table 1:** Hybrid schistosomes genetic profiles according to the rapid diagnostic (RD-PCR) mitochondrial cox1 analysis and by sequencing of the cox1 and ITS regions, respectively, from studies in Agboville (Côte d’Ivoire) and Corsica (c), France. The abbreviations Sb/Sb, Sh/Sb or Sh/Sh indicate that at the diagnostic sites two chromatogram peaks were visible after sequencing.

Study Location	RD-PCR Analysis	Sequence Analysis		Abbreviation
	cox1	cox 1 haplotypes	ITS2 alleles	
Agboville	*S. haematobium*	*S. haematobium*	*S. bovis + S. bovis*	Sh-Sb/Sb
	*S. bovis*	*S. bovis*	*S. haematobium + S. bovis*	Sb-Sh/Sb
	*S. bovis*	*S. bovis*	*S. haematobium+ S. haematobium*	Sb-Sh/Sh
	*S. haematobium*	*S. haematobium*	*S. haematobium + S. bovis*	Sh-Sh/Sb
Corsica	*S. bovis*	*S. bovis*	*S. haematobium + S. haematobium*	Sb-Sh/Sh^c^

Sb-Sh/Sh^c^: Hybrid schistosome from Corsica, France.

**Table 2:** Primer sets used in this work for *Schisto*-LAMP assays. For *S. mansoni, S. haematobium*, *S. bovis* and *Schistosoma* spp.: F3, forward outer primer; B3, backward outer primer; FIP, forward inner primer (comprising F1c and F2 sequences); BIP, backward inner primer (comprising B1c and B2 sequences); LF, loop forward primer; LB = loop backward primer. bp, base pairs.

*Schisto*-LAMP	Primer Sets	Sequence 5′→3′	Length (bp)	Ref.
*S. mansoni*	F3	TTATCGTCTATAGTACGGTAGG	22	[45]
	B3	ATACTTTAACCCCCACCAA	19	
	FIP	GCCAAGTAGAGACACAAACATCTT-TGGGTAAGGTAGAAAATGTTGT	47	
	BIP	AGAAGTGTTTAACTTGATGAAGGGG-AAACAAAACCGAAACCACTA	45	
*S. haematobium*	F3	CTTTCTAAGCCCGCGATA	18	[44]
	B3	GCGCATTACACTTGGTCT	18	
	FIP	TACCCCTAACTTCGTGGTCTCC-CCCCCTTATTTTAGGGTGC	41	
	BIP	CTCCCTATATAACATGGCGAGTAAG-ACTATGAAATCAGTGTTTTTCGG	48	
*S. bovis*	F3	TTCATTGTTAGGTTGCGT	18	[42]
	B3	TCTATATTCTACTCTAATCCCTCT	24	
	FIP	TCAGTATCATCTCAAACATCACACT-AGTAGTATGTTCTGTCTTAAGTT	48	
	BIP	TTTGTAGTACCTCTGGTTTACATCA-TTCACTCTCAGACTCTACAT	45	
	LF	ACTTAGACCATGAACATCAACCTAT	25	
	LB	TACTAAGTGAGAGTAATCGAACACC	25	
*Schistosoma* spp.	F3	TTGACCGGGGTACCTAGC	18	[42]
	B3	CGTGAATGGCAAGCCAAAC	19	
	FIP	ATCGCCCTTGGCAGATCAGG-CTGTCGTATGCCCTGATGG	39	
	BIP	ATATGCATGCAAATCCGCCCCG-CGGATCGCTTCAACAGTGTA	43	
	LF	CAGATCAGGCAACCCGAAAG	22	

## Data Availability

Not applicable.

## References

[1] Jones K.E., Patel N.G., Levy M.A., Storeygard A., Balk D., Gittleman J.L., Daszak P. (2008). Global trends in emerging infectious diseases. Nature.

[2] Huyse T., Webster B.L., Geldof S., Stothard J.R., Diaw O.T., Polman K., Rollinson D. (2009). Bidirectional introgressive hybridization between a cattle and human schistosome species. PLoS Pathog..

[3] Keesing F., Belden L.K., Daszak P., Dobson A., Harvell C.D., Holt R.D., Hudson P., Jolles A., Jones K.E., Mitchell C.E. (2010). Impacts of biodiversity on the emergence and transmission of infectious diseases. Nature.

[4] King K.C., Stelkens R.B., Webster J.P., Smith D.F., Brockhurst M.A. (2015). Hybridization in parasites: Consequences for adaptive evolution, pathogenesis, and public health in a changing world. PLoS Pathog..

[5] Webster J.P., Gower C.M., Knowles S.C.L., Molyneux D.H., Fenton A. (2016). One health—An ecological and evolutionary framework for tackling Neglected Zoonotic Diseases. Evol. Appl..

[6] Leger E., Webster J.P. (2017). Hybridizations within the genus *Schistosoma*: Implications for evolution, epidemiology and control. Parasitology.

[7] Colley D.G., Bustinduy A.L., Secor W.E., King C.H. (2014). Human schistosomiasis. Lancet.

[8] GBD 2016 Causes of Death Collaborators (2017). Global, regional, and national age-sex specific mortality for 264 causes of death, 1980-2016: A systematic analysis for the Global Burden of Disease Study 2016. Lancet.

[9] (2018). World Health Organization. https://www.who.int/news-room/fact-sheets/detail/schistosomiasis.

[10] Ohmae H., Sinuon M., Kirinoki M., Matsumoto J., Chigusa Y., Socheat D., Matsuda H. (2004). Schistosomiasis mekongi: From discovery to control. Parasitol. Int..

[11] Webster B.L., Southgate V.R., Timothy D., Littlewood J. (2006). A revision of the interrelationships of *Schistosoma* including the recently described *Schistosoma guineensis*. Int. J. Parasitol..

[12] Latif B., Heo C.C., Razuin R., Shamalaa D.V., Tappe D. (2013). Autochthonous human schistosomiasis, Malaysia. Emerg. Infect. Dis..

[13] Weyher A.H., Phillips-Conroy J.E., Fischer K., Weil G.J., Chansa W., Fischer P.U. (2010). Molecular identification of *Schistosoma mattheei* from feces of kinda (*Papio cynocephalus kindae*) and grayfoot baboons (*Papio ursinus griseipes*) in Zambia. J. Parasitol..

[14] De Bont J., Vercruysse J. (1997). The epidemiology and control of cattle schistosomiasis. Parasitol. Today.

[15] Webster B.L., Tchuem Tchuenté L.A., Jourdane J., Southgate V.R. (2005). The interaction of *Schistosoma haematobium* and *S. guineensis* in Cameroon. J. Helminthol..

[16] Southgate V.R., van Wijk H.B., Wright C.A. (1976). Schistosomiasis at Loum, Cameroun; *Schistosoma haematobium*, *Schistosoma haematobium*, *S. intercalatum* and their natural hybrid. Z. Parasitenkd..

[17] Webster B.L., Southgate V.R., Tchuem Tchuenté L.-A. (2003). Isoenzyme analysis of *Schistosoma haematobium*, *S. intercalatum* and their hybrids and occurrences of natural hybridization in Cameroon. J. Helminthol..

[18] Webster B.L., Alharbi M.H., Kayuni S., Makaula P., Halstead F., Christiansen R., Juziwelo L., Stanton M.C., LaCourse E.J., Rollinson D. (2019). Schistosome interactions within the *Schistosoma haematobium* group, Malawi. Emerg. Infect. Dis..

[19] Boissier J., Grech-Angelini S., Webster B.L., Allienne J.F., Huyse T., Mas-Coma S., Toulza E., Barré-Cardi H., Rollinson D., Kincaid-Smith J. (2016). Outbreak of urogenital schistosomiasis in Corsica (France): An epidemiological case study. Lancet Infect. Dis..

[20] De la Torre-Escudero E., Pérez-Sánchez R., Manzano-Román R., Oleaga A. (2017). *Schistosoma bovis*-host interplay: Proteomics for knowing and acting. Mol. Biochem. Parasitol..

[21] Kincaid-Smith J., Rey O., Toulza E., Berry A., Boissier J. (2017). Emerging Schistosomiasis in Europe: A need to quantify the risks. Trends Parasitol..

[22] Oey H., Zakrzewski M., Gravermann K., Young N.D., Korhonen P.K., Gobert G.N., Nawaratna S., Hasan S., Martínez D.M., You H. (2019). Whole-genome sequence of the bovine blood fluke *Schistosoma bovis* supports interspecific hybridization with *S. haematobium*. PLoS Pathog..

[23] Savassi B.A.E.S., Mouahid G., Lasica C., Mahaman S.D.K., Garcia A., Courtin D., Allienne J.F., Ibikounlé M., Moné H. (2020). Cattle as natural host for *Schistosoma haematobium* (Bilharz, 1852) Weinland, 1858 x *Schistosoma bovis* Sonsino, 1876 interactions, with new cercarial emergence and genetic patterns. Parasitol. Res..

[24] De Bont J., Vercruysse J., Southgate V.R., Rollinson D., Kaukas A. (1994). Cattle Schistosomiasis in Zambia. J. Helminthol..

[25] Charlier J., van der Voort M., Kenyon F., Skuce P., Vercruysse J. (2014). Chasing helminths and their economic impact on farmed ruminants. Trends Parasitol..

[26] Angora E.K., Allienne J.F., Rey O., Menan H., Touré A.O., Coulibaly J.T., Raso G., Yavo W., N’Goran E.K., Utzinger J. (2020). High prevalence of *Schistosoma haematobium* x *Schistosoma bovis* hybrids in schoolchildren in Côte d’Ivoire. Parasitology.

[27] Pennance T., Allan F., Emery A., Rabone M., Cable J., Garba A.D., Hamidou A.A., Webster J.P., Rollinson D., Webster B.L. (2020). Interactions between *Schistosoma haematobium* group species and their *Bulinus* spp. intermediate hosts along the Niger River Valley. Parasites Vectors.

[28] Soentjens P., Cnops L., Huyse T., Yansouni C., De Vos D., Bottieau E., Clerinx J., Van Esbroeck M. (2016). Diagnosis and clinical management of *Schistosoma haematobium-Schistosoma bovis* hybrid infection in a cluster of travelers returning from Mali. Clin. Infect. Dis..

[29] Holtfreter M.C., Moné H., Müller-Stöver I., Mouahid G., Richter J. (2014). *Schistosoma haematobium* infections acquired in Corsica, France, August 2013. Eurosurveillance.

[30] Moné H., Holtfreter M.C., Mouahid G., Richter J. (2016). Difficulties in Schistosomiasis assessment, Corsica, France. Emerg. Infect. Dis..

[31] Bärenbold O., Raso G., Coulibaly J.T., N’Goran E.K., Utzinger J., Vounatsou P. (2017). Estimating sensitivity of the Kato-Katz technique for the diagnosis of *Schistosoma mansoni* and hookworm in relation to infection intensity. PLoS Negl. Trop. Dis..

[32] Chuah C., Gobert G.N., Latif B., Heo C.C., Leow C.Y. (2019). Schistosomiasis in Malaysia: A review. Acta Trop..

[33] Weerakoon K.G.A.D., Gobert G.N., Cai P., McManus D.P. (2015). Advances in the diagnosis of human schistosomiasis. Clin. Microbiol. Rev..

[34] Hinz R., Schwarz N.G., Hahn A., Frickmann H. (2017). Serological approaches for the diagnosis of schistosomiasis—A review. Mol. Cell. Probes.

[35] McManus D.P., Dunne D.W., Sacko M., Utzinger J., Vennervald B.J., Zhou X.N. (2018). Schistosomiasis. Nat. Rev. Dis. Prim..

[36] Weerakoon K.G., Gordon C.A., McManus D.P. (2018). DNA diagnostics for schistosomiasis control. Trop. Med. Infect. Dis..

[37] Schols R., Carolus H., Hammoud C., Mulero S., Mudavanhu A., Huyse T. (2019). A rapid diagnostic multiplex PCR approach for xenomonitoring of human and animal schistosomiasis in a “One Health” context. Trans. R. Soc. Trop. Med. Hyg..

[38] Notomi T., Okayama H., Masubuchi H., Yonekawa T., Watanabe K., Amino N., Hase T. (2000). Loop-mediated isothermal amplification of DNA. Nucleic Acids Res..

[39] Wong Y.P., Othman S., Lau Y.L., Radu S., Chee H.Y. (2018). Loop-mediated isothermal amplification (LAMP): A versatile technique for detection of micro-organisms. J. Appl. Microbiol..

[40] Mori Y., Notomi T. (2020). Loop-mediated isothermal amplification (LAMP): Expansion of its practical application as a tool to achieve universal health coverage. J. Infect. Chemother..

[41] Avendaño C., Patarroyo M.A. (2020). Loop-mediated isothermal amplification as point-of-care diagnosis for neglected parasitic infections. Int. J. Mol. Sci..

[42] Fernández-Soto P., Avendaño C., Sala-Vizcaíno A., Crego-Vicente B., Febrer-Sendra B., García-Bernalt Diego J., Oleaga A., López-Abán J., Vicente B., Patarroyo M.A. (2020). Molecular markers for detecting *Schistosoma* species by Loop-Mediated Isothermal Amplification. Dis. Markers.

[43] Lewis F.A., Liang Y.S., Raghavan N., Knight M. (2008). The NIH-NIAID schistosomiasis resource center. PLoS Negl. Trop. Dis..

[44] Gandasegui J., Fernández-Soto P., Carranza-Rodríguez C., Pérez-Arellano J.L., Vicente B., López-Abán J., Muro A. (2015). The rapid-heat LAMPellet method: A potential diagnostic method for human urogenital schistosomiasis. PLoS Negl. Trop. Dis..

[45] Fernández-Soto P., Gandasegui Arahuetes J., Sánchez Hernández A., López Abán J., Vicente Santiago B., Muro A. (2014). A Loop-Mediated Isothermal Amplification (LAMP) assay for early detection of *Schistosoma mansoni* in stool samples: A diagnostic approach in a murine model. PLoS Negl. Trop. Dis..

[46] Pennance T., Ame S.M., Amour A.K., Suleiman K.R., Allan F., Rollinson D., Webster B.L. (2018). Occurrence of *Schistosoma bovis* on Pemba Island, Zanzibar: Implications for urogenital schistosomiasis transmission monitoring. Parasitology.

[47] Webster B.L., Diaw O.T., Seye M.M., Webster J.P., Rollinson D. (2013). Introgressive hybridization of *Schistosoma haematobium* group species in Senegal: Species barrier break down between ruminant and human schistosomes. PLoS Negl. Trop. Dis..

[48] Léger E., Garba A., Hamidou A.A., Webster B.L., Pennance T., Rollinson D., Webster J.P. (2016). Introgressed animal schistosomes *Schistosoma curassoni* and *S. bovis* naturally infecting humans. Emerg. Infect. Dis..

[49] Huyse T., Van Den Broeck F., Hellemans B., Volckaert F.A.M., Polman K. (2013). Hybridisation between the two major African schistosome species of humans. Int. J. Parasitol..

[50] Catalano S., Sène M., Diouf N.D., Fall C.B., Borlase A., Léger E., Bâ K., Webster J.P. (2018). Rodents as natural hosts of zoonotic *Schistosoma* species and hybrids: An epidemiological and evolutionary perspective from West Africa. J. Infect. Dis..

[51] Catalano S., Léger E., Fall C.B., Borlase A., Diop S.D., Berger D., Webster B.L., Faye B., Diouf N.D., Rollinson D. (2020). Multihost transmission of *Schistosoma mansoni*. Emerg. Infect. Dis..

[52] Pena H.B., De Souza C.P., Simpson A.J.G., Pena S.D.J. (1995). Intracellular promiscuity in *Schistosoma mansoni*: Nuclear transcribed DNA sequences are part of a mitochondrial minisatellite region. Proc. Natl. Acad. Sci. USA.

[53] Gandasegui J., Fernández-Soto P., Muro A., Simões Barbosa C., Lopes de Melo F., Loyo R., de Souza Gomes E.C. (2018). A field survey using LAMP assay for detection of *Schistosoma mansoni* in a low-transmission area of schistosomiasis in Umbuzeiro, Brazil: Assessment in human and snail samples. PLoS Negl. Trop. Dis..

[54] Fernández-Soto P., Gandasegui J., Rodríguez C.C., Pérez-Arellano J.L., Crego-Vicente B., García-Bernalt Diego J., López-Abán J., Vicente B., Muro A. (2019). Detection of *Schistosoma mansoni*-derived DNA in human urine samples by loop-mediated isothermal amplification (LAMP). PLoS ONE.

[55] Le Govic Y., Kincaid-Smith J., Allienne J.F., Rey O., de Gentile L., Boissier J. (2019). *Schistosoma haematobium- Schistosoma mansoni* hybrid parasite in migrant boy, France, 2017. Emerg. Infect. Dis..

[56] Zhao G.H., Mo X.H., Zou F.C., Li J., Weng Y.B., Lin R.Q., Xia C.M., Zhu X.Q. (2009). Genetic variability among *Schistosoma japonicum* isolates from different endemic regions in China revealed by sequences of three mitochondrial DNA genes. Vet. Parasitol..

[57] Vilas R., Criscione C.D., Blouin M.S. (2005). A comparison between mitochondrial DNA and the ribosomal internal transcribed regions in prospecting for cryptic species of platyhelminth parasites. Parasitology.

[58] Webster B.L., Culverwell C.L., Khamis I.S., Mohammed K.A., Rollinson D., Stothard J.R. (2013). DNA barcoding of *Schistosoma haematobium* on Zanzibar reveals substantial genetic diversity and two major phylogenetic groups. Acta Trop..

[59] Nolan M.J., Cribb T.H. (2005). The use and implications of ribosomal DNA sequencing for the discrimination of digenean species. Adv. Parasitol..

[60] Cutmore S.C., Bennett M.B., Cribb T.H. (2010). *Staphylorchis cymatodes* (*Gorgoderidae: Anaporrhutinae*) from carcharhiniform, orectolobiform and myliobatiform elasmobranchs of Australasia: Low host specificity, wide distribution and morphological plasticity. Parasitol. Int..

[61] Kane R.A., Rollinson D. (1994). Repetitive sequences in the ribosomal DNA internal transcribed spacer of *Schistosoma haematobium, Schistosoma intercalatum* and *Schistosoma mattheei*. Mol. Biochem. Parasitol..

[62] Dvořák J., Vaňáčová Š., Hampl V., Flegr J., Horák P. (2002). Comparison of European *Trichobilharzia* species based on ITS1 and ITS2 sequences. Parasitology.

[63] Van Herwerden L., Blair D., Agatsuma T. (1998). Intra- and inter-specific variation in nuclear ribosomal internal transcribed spacer 1 of the *Schistosoma japonicum* species complex. Parasitology.

[64] Kane R.A., Rollinson D. (1998). Comparison of the intergenic spacers and 3’ end regions of the large subunit (28S) ribosomal RNA gene from three species of *Schistosoma*. Parasitology.

[65] El Bali L., Diman A., Bernard A., Roosens N.H.C., Dekeersmaecker S.C.J. (2014). Comparative study of seven commercial kits for human DNA extraction from urine samples suitable for DNA biomarker-based public health studies. J. Biomol. Tech..

